# Removal of syndesmotic screws – is sonography a precise and efficient method of guidance?

**DOI:** 10.1186/s12891-026-09569-4

**Published:** 2026-02-06

**Authors:** Christoph Eckstein, Dmytro Oliinyk, Raffael Peteler, Claus-Philipp Stefan, Paul Schmitz

**Affiliations:** 1 Department of Trauma Surgery, University Medical Centre Regensburg, Caritas St. Josef, Landshuter Strasse 65, Regensburg, 93053 Germany; 2 Department of Plastic and Reconstructive Surgery, University Medical Centre Regensburg, Franz-Josef-Strauss-Allee 11, Regensburg, 93053 Germany

**Keywords:** Syndesmotic injury, Ankle joint fractures, Syndesmotic screws, Sonography, Implant removal, Screw removal

## Abstract

**Background:**

Syndesmotic injuries can lead to ankle instability. They are treated with syndesmotic screws or suture device. The necessity for screw removal remains a topic of debate. When removal is performed, it usually occurs 2 to 3 months post-operation. In such cases, the screw is typically visualized flouroscopically before removal. We describe an ultrasound-guided procedure for screw removal that avoids radiation exposure and has minimal infrastructural requirements.

**Method:**

In this prospective cohort study, Cohort 1 included 26 screws (18 patients) that were removed under both fluoroscopic and ultrasound guidance. This allowed for the assessment of the accuracy of ultrasound compared to fluoroscopic visualization. In Cohort 2, 22 screws (17 patients) were removed solely under ultrasound guidance to evaluate the practical applicability of the method.

**Results:**

A total of 35 patients were included into our study, eighteen in the first and seventeen in the second cohort respectively. In Cohort 1, the mean distance between the fluoroscopic and ultrasound measurement points of the screw heads was 2.9 mm. The mean radiation dose was 1.4 cGy/cm², and the mean operation duration was 15.2 min. Cohort 2 had a mean operative duration of 10.3 min. There were no significant differences in baseline characteristics between the groups. Radiation exposure was not present in Cohort 2. In Cohort 1, the ultrasound-guided screw localization revealed high accuracy, with the threshold < 5 mm (*p* = 0.040). We did not identify any significant predictors for the screw localization accuracy. The duration of the surgical procedure was similar in both groups. In the entire pooled cohort, a longer distance from skin to screw was associated with a longer surgery duration (ρ = 0.419, *p* = 0.012). Skin-to-screw distance was found to be the only independent predictor of surgery duration (*p* = 0.009).

**Conclusion:**

Ultrasound-guided localisation and removal of syndesmotic screws demonstrate comparable accuracy compared to fluoroscopy, with the additional benefits of lower infrastructural requirements and associated costs. Furthermore, this method has a similar operative time to fluoroscopy and eliminates radiation exposure, supporting its feasibility as an efficient and safe alternative for syndesmotic screw removal.

**Trial registration:**

In accordance with the Declaration of Helsinki the study protocol was approved by the Ethics Review Board of the University of Regensburg, Protocol number 21-2204-101.

## Background

Fractures of the ankle joint are among the most common injuries of the lower extremity [1]. Up to 20% of these fractures and 0.5% of ankle sprains are associated with a surgically relevant injury to the syndesmotic complex [2, 3]. These injuries are treated by transfixing the distal tibiofibular joint in an orthograde position using a screw or suture device until the syndesmotic ligament complex has healed [4]. The literature shows better outcomes for suture devices [5]. However, there are biomechanical studies that demonstrate that a suture device alone does not provide sufficient stability [6, 7]. Therefore, some authors still consider the transsyndesmotic screw as the “gold standard” [8]. The usual diameter of the screws used is 3.5–4.5 mm [9]. There is currently no consensus on whether one or two screws should be implanted or whether the screws should have tri- or quadricortical purchase [10, 11]. It is assumed that after two to three months, the ligaments have healed and fixation is no longer needed [12]. Typically, the screws are removed before weight-bearing is initiated, as they interfere with the movements of the fibula. These movements are likely responsible for screw fractures, which occur with a frequency of 2.1% to 14.3% [13]. The removal of screws is still a topic of debate in many studies, which indicate that retaining screws does not lead to worse outcomes in terms of function or pain [14]. Given the surgical risks and the economic burden on the healthcare system, routine removal of screws is therefore not recommended [15, 16]. However, the evidence is not entirely conclusive. Studies report poorer outcomes, increased stiffness and reduced mobility and activity levels with retained screws [10, 17]. Moreover, it has been hypothesized that the removal of the screws may result in spontaneous correction of malreduction of the distal tibiofibular joint [18]. In cases of intraosseous screw breakage, bony erosions can cause pain [19]. In our practice, we opt to remove screws in younger patients and those with high activity levels. Fluoroscopic guidance is generally utilized during the removal process, exposing both patients and surgeons to ionizing radiation and necessitating specialized infrastructure. This study seeks to assess whether ultrasound- guide removal of syndesmotic screws can achieve comparable diagnostic accuracy and procedural time to fluoroscopy, thereby offering evidence for a radiation-free and more accessible alternative within orthopedic practice.

## Methods

The primary aim of the current study is to determine whether ultrasound-guided localization of the syndesmotic screw has comparable accuracy to fluoroscopy-assisted procedures. Furthermore, we aim to investigate whether this can be achieved in a similar timeframe.

### Patients

**Fig. 1:**
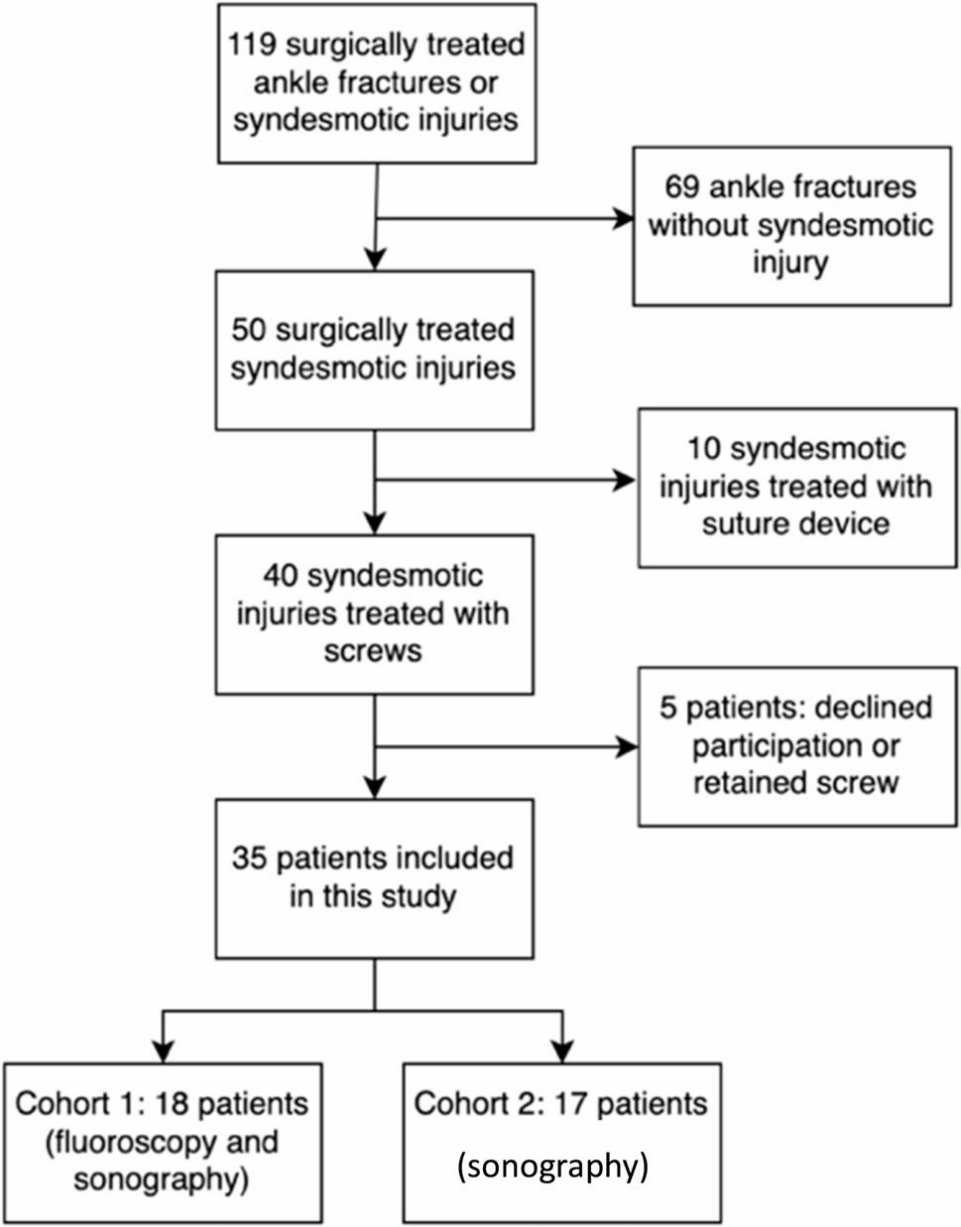
The participant flow chart diagram illustrates the identification of the study participants

Our prospective cohort study was conducted between January 2022 and June 2024. During this period, 119 ankle fractures were surgically treated at our clinic. Of these, 40 patients received a syndesmotic screw, and 10 patients received a suture device due to an injury to the syndesmotic complex. Only patients who underwent screw fixation for syndesmotic injury and subsequently had surgical screw removal were included in the study (Fig. 1). The patients received only screws that were 3.5 mm in diameter. Exclusion criteria were unwillingness to participate in the study or refusal of screw explantation. All patients received detailed written surgical information about the operation with the necessary forms. This was followed by information about the study and the visualization of the screws. The study was performed in accordance with the Declaration of Helsinki and the study was approved by the Ethics Review Board of the University of Regensburg, Protocol number 21–2204-101. Every participant provided informed consent to participate in the study. Every participant provided written informed consent for publication of the manuscript including any accompanying images or data contained within the manuscript that may directly or indirectly disclose their identity. This research received no specific grant from any funding agency in the public, commercial, or not-for-profit sectors.

### Ultrasonography

**Fig. 2:**
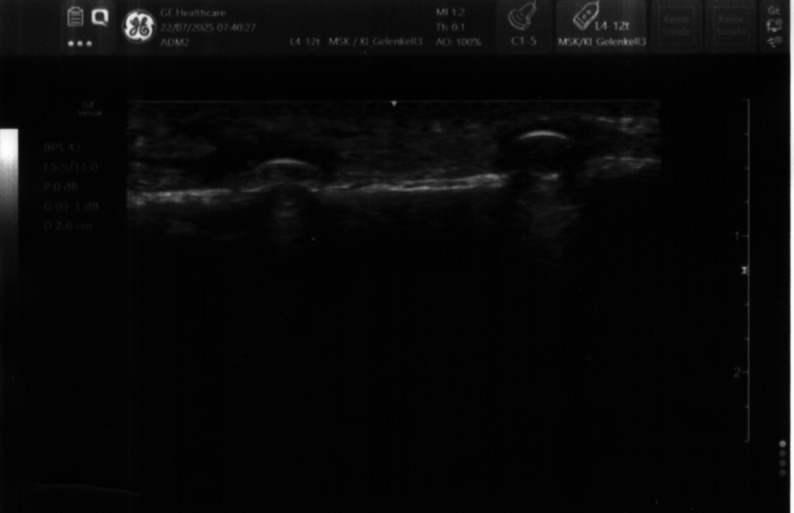
The image shows the longitudinal sonographic view of two syndesmotic screws in situ, appearing as hyperechoic structures with comet-tail artifacts. The image corresponds to a 49-year-old female patient (BMI 26.1) with a history of ankle sprain and syndesmotic injury treated by syndesmotic screw fixation

Ultrasonography of the syndesmotic screw was performed prior to operative removal using an ultrasound machine (GE, Venue R2) with a linear probe (9 L, 4.5- 9.0 MHz) and a thin layer of acoustic coupling gel. The syndesmotic screw causes a resonance artifact during ultrasound examination, appearing as hyperechoic with reverberation (so-called “comet tail”). In contrast, bone is detected as hyperechoic with an underlying acoustic shadow (Fig. 2) [20]. A brief summary of the sonographic appearances of the examined structures is displayed in Table 1.

**Table 1:** Sonographic appearance of the examined structures

Structure	Sonographic Appearance [20, 21]
Epidermis	Hyperechoic
Subcutis	Hypoechoic fat and hyperechoic fibrous tissue, Fig. 2
Bone	Hyperechoic with an underlying acoustic shadow, Fig. 2
Screw	Hyperechoic with reverberation artifacts (“comet tail”), Fig. 2
Plate	Hyperechoic with reverberation artifacts, Fig. 3

**Fig. 3:**
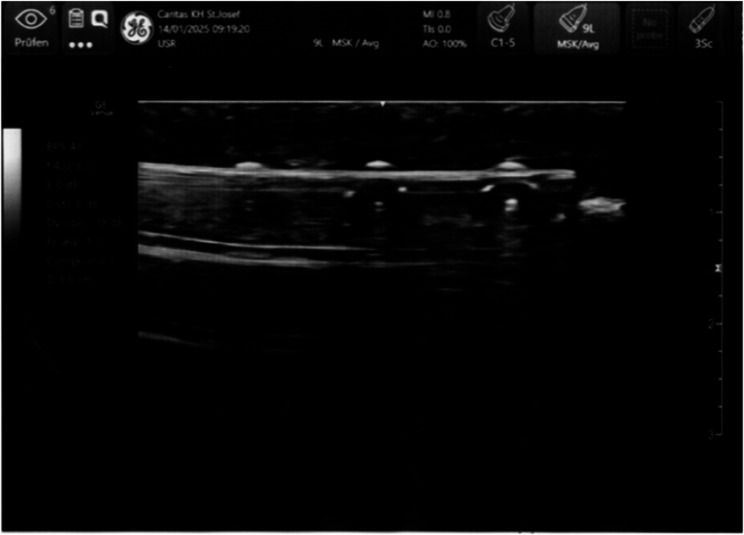
Sonographic image of a plate with screws in the longitudinal plane. The individual screws, the plate and the bone can be clearly distinguished. The image corresponds to a 59-year-old female patient (BMI 22.8) with ankle fracture (type Weber B) and syndesmotic injury

In cases of plate osteosynthesis, individual screw heads can be detected. In correlation with X-ray images of the fracture treatment, the position of the syndesmotic screw can thus be determined and marked (Fig. 3).

**Fig. 4:**
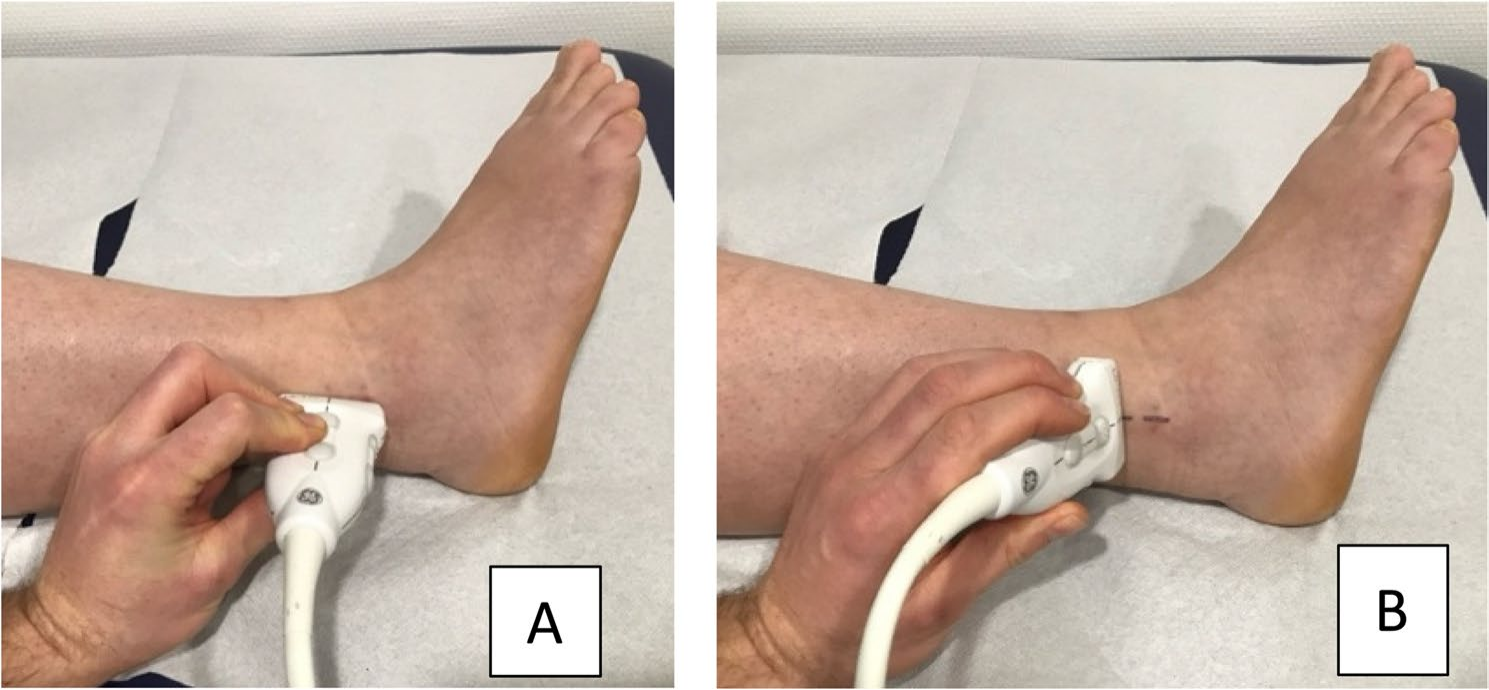
Preoperative ultrasound-guided localization of syndesmotic screws for surgical removal. The two clinical images demonstrate correct positioning of the ultrasound transducer over the lateral ankle: **A** in the longitudinal plane; **B** in the transverse plane for orthogonal confirmation and precise cross-referencing of the screw location. Skin marking is performed at the intersection of the two planes to guide incision. The images correspond to a 52-year-old female patient (BMI 33.2) with a history of ankle sprain and syndesmotic injury treated by syndesmotic screw fixation.

Patients were examined in a supine position. The distal fibula at the site of the surgical scar was displayed in the longitudinal plane, and the location of the screw was marked using a skin marker. Subsequently, the screw was visualized in the transverse plane via ultrasound, and a marking was made (Fig. 4). The intersection point of both lines indicated the position of the screw. The examinations were performed by six different residents who later carried out the surgical removal under the supervision of a senior trauma surgeon.

**Fig. 5:**
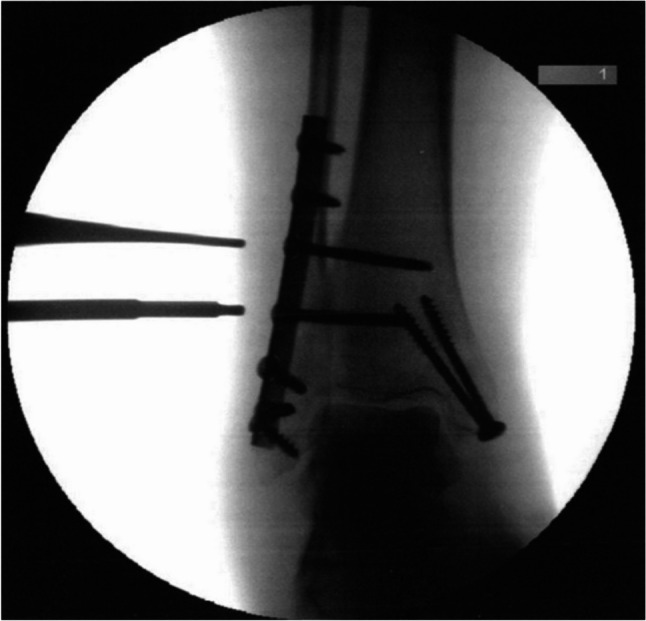
Intraoperative fluoroscopic vizualization oft two syndesmotic screws. The image corresponds to a 57-year-old female patient (BMI 23.4) with trimalleolar ankle fracture

**Fig. 6:**
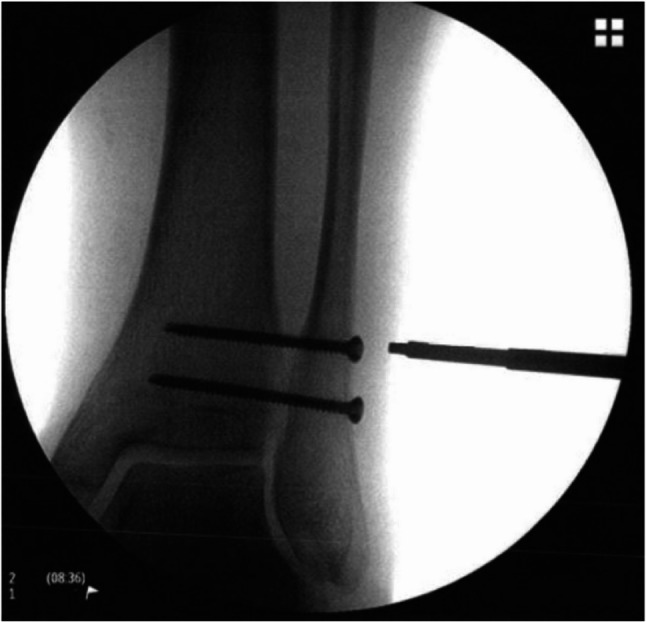
Intraoperative fluoroscopic visualization of two syndesmotic screws. The image corresponds to a 65-year-old male patient (BMI 33.8) with a Maisonneuve fracture treated by syndesmotic screw fixation

Two consecutive cohorts were formed. In Cohort 1, the screw position was additionally visualized intraoperatively using radiological methods in an anterior-posterior view (Ziehm, Vision RFD) and marked (Figs. 5 and 6). The distance (in millimeters) between the two measurement points (ultrasound and radiology) was measured. The measurements were compared with a predefined threshold of 5 mm. This threshold was chosen because it corresponds to the diameter of the screw heads that had to be displayed. The objective was to analyze the accuracy of ultrasound visualization compared to radiological visualization.

In Cohort 2, radiological control of the screw position was omitted. Visualization was performed solely with ultrasound assistance. A fluoroscope (Ziehm, Vision RFD) was kept on standby but not used. This approach aimed to test the practicality of the method presented here without endangering the patient.

### Surgical technique

After prior desinfection, local anesthesia was administered at the intersection point of the markings (Lidocaine 1%, 8 ml). Following this, disinfection and sterile draping of the surgical area were performed. A skin incision of approximately 0.5 cm was then made through the scar of the previous surgical incision. Blunt dissection was carried out with scissors to reach deeper tissues, the screw head was palpated, and the screw was removed using a screwdriver. Wound irrigation followed, and the wound was closed with sutures (Ethicon, Ethilon 3 − 0) and a sterile dressing.

### Postoperative treatment

Postoperative pain management was conducted with oral analgesics. Routine radiological control was performed in all cohorts after the removal of the syndesmotic screw. Gradual weight-bearing up to full weight-bearing was recommended. A final clinical-radiological assessment took place six weeks after the removal of the syndesmotic screw.

### Statistics

Shapiro-Wilk test was used to determine whether continuous variables were normal because of the relatively small sample sizes (less than 30 per cohort), the. Non-parametric tests were used throughout because the majority of the variables were not normally distributed. For continuous or ordinal variables (such as surgery duration, surgery time per screw, or number of screws), the Mann–Whitney U test was used to compare the two cohorts. The Fisher’s exact test, which is suitable for small sample sizes and binary data, was used to compare categorical variables (such as sex, side, plating, infection and obesity). In cohort 1, where intraoperative sonographic and fluoroscopic markings were recorded, the precision of ultrasound-guided screw localization was evaluated. In order to find the reasons related to the inaccuracies in this measurement (e.g., BMI, infection, presence of a plate, skin-to-screw distance), Mann–Whitney U tests were carried for categorical variables, and the correlation of Spearman was used for continuous ones. The estimated accuracy of ultrasound-guided screw localization was compared to a predefined clinical threshold of 5 mm (diameter of the heads of the implanted screws) by one-sample Wilcoxon signed-rank test (distance between sonographic and fluoroscopic markings was the measurement).

Additionally, a bootstrapped multivariate linear regression model was built for the dependent variable surgery duration to identify predictors of the screw localization accuracy. Independent variables were cohort, age, obesity, skin-to-screw distance, infection status, and the presence of a plate. The number of resamples in the bootstrapping process was set at 1.000 in order to get the most accurate estimates and to construct confidence intervals, thus the effects of non-normal distribution and outliers are minimized.

Moreover, a separate bootstrapped multivariate linear regression was conducted to estimate the possible predictors of surgical performance (measured by surgery duration) with the same list of covariates. Two-sided p-values of less than 0.05 were deemed to indicate significance in all the tests. In the bootstrap regression, the indication of being significant was if the 95% confidence interval did not cross zero. Statistic analysis was carried out using IBM SPSS Statistics Version 29.0.2.0 (20).

## Results

### Descriptive analysis

In our study two cohorts with a total of 35 patients have been analyzed, 18 patients within the first cohort and 17 patients within the second, respectively. After preliminary analysis for normality of the data differences between the cohorts have been assessed (Table 1). No statistically significant differences in age were found between the groups (Cohort 1: median 54.1 years [range: 16.0–81.0], Cohort 2: 48.6 years [21.0–75.0]; *p* = 0.298, Mann-Whitney U test). Sex distribution was comparable (Cohort 1: 38.9% female; Cohort 2: 35.3% female; *p* > 1.000, Fisher’s exact test). While the presence of a plate was more common in Cohort 2 (82.4%) than in Cohort 1 (55.6%), this difference did not reach statistical significance (*p* = 0.146, Fisher’s exact test). No significant differences between cohorts regarding the side affected (right vs. left: *p* = 0.733), presence of infection (*p* = 0.603), or obesity defined as BMI > 30 kg/m² (*p* = 0.738) were found. Median BMI values were also not significantly different (Cohort 1: 28.5 [22.6–35.3]; Cohort 2: 31.2 [22.3–41.0]; *p* = 0.235). The number of screws implanted was similar between cohorts (Cohort 1: 1.4 [1.0–2.0]; Cohort 2: 1.3 [1.0–2.0]; *p* = 0.365). Furthermore, there were no significant differences found in time from osteosynthesis to implant removal (Cohort 1: 6.8 weeks; Cohort 2: 6.95 weeks; *p* = 0.690), distance from skin to the screw (Cohort 1: 7.9 mm; Cohort 2: 8.5 mm; *p* = 0.653), duration of surgery (Cohort 1: 15.1 min; Cohort 2: 10.3 min; *p* = 0.154), or duration of surgery per screw (Cohort 1: 11.1 min; Cohort 2: 8.5 min; *p* = 0.297). The mean dose exposure (Cohort 1) was recorded at 1.4 cGy/cm² (range 0.2–4.9, SD 1.2). The dose-area product of radiation was absent in Cohort 2. The average distance between the screw localization points using fluoroscopic and ultrasound was 2.89 mm (range: 0.0–13.0) in Cohort 1; this parameter was not applicable for Cohort 2, which did not use fluoroscopic guidance.

### Accuracy of the method – primary end-points

In order to verify the accuracy of screw localization by ultrasound, a one-sample Wilcoxon signed-rank test was conducted comparing the distances from the observed screw marks made with the sonography and the fluoroscopy to a reference value of 5 mm, which was the diameter of the head of the screws used in the study. So, a one-sample Wilcoxon signed-rank test indicated that the median difference (1.0 mm) was significantly lower than the 5 mm threshold (*p* = 0.04).

Further, we decided to explore potential factors that may interfere with the surrogate parameter of the accuracy, namely with the distance between our fluoroscopic and sonography markings. In this way, the presence of osteosynthesis plating did not show any association with the accuracy of the method (Mann-Whitney U = 33.5; *p* = 0.558), nor did obesity—defined as BMI > 30 kg/m² (Mann-Whitney U 36.0; *p* = 0.818)—demonstrate a significant association with ultrasound localization accuracy, as measured by the distance between fluoroscopic and sonographic markings in Cohort 1. Additionally, there was no significant correlation between the skin-to-screw distance and ultrasound localization accuracy, as measured by the distance between sonographic and fluoroscopic markings (Spearman’s ρ = 0.284, *p* = 0.254). However, there was a weak trend for a lesser accuracy of the presented method with an increasing number of syndesmotic screws (Spearman’s ρ = − 0.438, *p* = 0.069). In the robust, bootstrapped multivariate linear regression model assessing the accuracy of screw localization (measured by the distance between sonographic and fluoroscopic markings), no statistically significant predictors of accuracy were identified. For example, the distance from the skin to the screw had reached a p-value of 0.184 with a 95% CI ranging from − 0.236 to 1.959.

### Group comparisons – secondary end-point analysis

A secondary end-point analysis between the cohorts and in the pooled cohort assessing surgery time has been carried out. We did not find any statistically significant difference in total surgery duration between Cohort 1 and Cohort 2 (Mann–Whitney U = 110.0, *p* = 0.154), nor in surgery duration per screw (Mann–Whitney U = 121.5, *p* = 0.297). A correlation analysis of the total surgery duration showed a weak tendency for a positive correlation between a total surgery duration and the number of screws (Spearman’s ρ = 0.321, *p* = 0.060), a significant correlation with the distance from the skin surface to the screw (Spearman’s ρ = 0.419, *p* = 0.012 and Spearman’s ρ = 0.468, *p* = 0.005 for the time per screw), a weak tendency for the presence of an infection (Spearman’s ρ = 0.304, *p* = 0.076). In the robust, bootstrapped multivariate regression model predicting surgery duration, the distance from the skin to the screw was significantly associated with longer surgery times (*p* = 0.009). The presence of an infection showed a weak trend toward longer surgery duration but did not reach statistical significance (*p* = 0.110). The cohort variable (Cohort 1 vs. Cohort 2) was not significantly associated with surgery duration (*p* = 0.161) neither (Table 2).

**Table 2:** Patients’ characteristics and differences between cohorts

Variable	Cohort 1	Cohort 2	Fisher´s exact Test, *p*-value	Mann-Whitney U Test, *p*-value
Age	54.1 (16.0–81.0, SD = 54.1)	48.6 (21.0–75.0, SD = 15.8)	NA	*p* = 0.298
Sex				
Female	7 (38.9%)	6 (35.3%)	*p > 1.000*	NA
Male	11 (61.1%)	11 (64.7%)		
Presence of a plate				
Yes	10 (55.6%)	14 (82.4%)	*p = 0.146*	NA
No	8 (44.4%)	3 (17.6%)		
Affected site				
Right	8 (44.4%)	6 (35.3%)	*p = 0.733*	NA
Left	10 (55.6%)	11 (64.7%)		
Presence of infection				
Yes	3 (16.7%)	1 (5.9%)	*p = 0.603*	NA
No	15 (83.3%)	16 (94.1%)		
Obesity with BMI > 30 kg/m^2^				
Yes	7 (38.9%)	8 (47.1%)	*p = 0.738*	NA
No	11 (61.1%)	9 (52.9%)		
BMI	28.5 (22.6–35.3, SD = 28.5)	31.2 (22.3–41.0, SD = 5.9)	NA	*p* = 0.235
Number of screws	1.4 (1.0–2.0, SD = 0.5)	1.3 (1.0–2.0, SD = 0.47)	NA	*p* = 0.365
Time from osteosynthesis to removal to the material in *weeks*	6.8 (6.0–9.5.0.5, SD = 1.04)	6.95 (6.0–11.1.0.1, SD = 1.3)	NA	*p* = 0.690
Distance from skin to the screw in *mm*	7.9 (5.0–15.0, SD = 2.8)	8.5 (3.5–14.0, SD = 3.5)	NA	*p* = 0.653
Duration of surgery in *min*	15.1 (3.0–28.0, SD = 8.99)	10.3 (5.0–24.0, SD = 5.6)	NA	*p* = 0.154
Duration of surgery per screw in *min*	11.1 (3.0–28.0, SD = 6.9)	8.5 (3.0–24.0, SD = 5.2)	NA	*p* = 0.297
Total	18 (100%)	17 (100%)	NA	NA

## Discussion

Ultrasound is commonly used for the diagnosis of soft tissues. Increasingly, it is also being utilized in the assessment of bone, particularly in the context of pediatric fracture diagnosis and monitoring [22]. Additional applications in this regard include intraoperative control of fracture reduction, assistance in locating the correct entry point for an intramedullary nail, and visualization of locking screws during material removal [23, 24]. Wu et al. described a sonographically guided procedure for dynamization of intramedullary nails in 2013, where the corresponding screws were preoperatively marked using ultrasound and removed after local infiltration with an anesthetic. In their study, the mean depth of the screws was reported to be 34 mm, significantly more than in our study. Here, the thickness of the soft tissue mantle averaged 7.9 mm (Cohort 1) and 8.5 mm (Cohort 2). The difference can be explained by the fact that in Wu et al.‘s study, most cases involved the removal of locking screws from femoral intramedullary nails, where the soft tissue mantle is more pronounced. The mean operative time reported in their publication was 3.5 min, which is considerably shorter than in our study (Cohort 1: 15.2 min and Cohort 2: 10.3 min). However, in Wu et al.‘s work, only one screw was removed, and the procedures were performed by two experienced surgeons [25]. In our study, two screws had to be removed in 13 patients (37.1%), and the procedures were conducted by six different residents under the supervision of a senior trauma surgeon. Both points also explain the pronounced fluctuations in the operation time, which range from 3 min to 28 min. In this context, it is noteworthy that the duration of the surgery did not show a statistically significant difference between the two cohorts (*p* = 0.154). Thus, the method described here did not result in a longer operative time. Only the thickness of the soft tissue envelope demonstrated a statistically significant correlation with the duration of the procedure (*p* = 0.009). This seems reasonable, as the screws are more difficult to locate in a more extensive soft tissue mantle. Regarding the number of screws (*p* = 0.235), the presence of a plate (*p* = 0.763), or the BMI (*p* = 0.534), no statistically significant association could be established. These factors do not appear to influence the duration of the procedure. This is particularly relevant for the practicality of the method, as a longer operative time is associated with increased costs.

The measurements in Cohort 1 demonstrated a mean difference of 2.9 mm between fluoroscopic and ultrasonographic measurements. This value was below our cutoff of 5 mm, which corresponds to the diameter of the depicted screw heads. We believe that the method described here exhibits comparable accuracy to radiographic imaging. The latter also showed no statistically significant correlation with the presence of a plate (*p* = 0.558), obesity (*p* = 0.818), or the thickness of the soft tissue envelope (*p* = 0.254). This underscores that the described method can localize the screws precise under various conditions—without the risk of radiation exposure.

In the literature, wound infections following screw removal are reported with a frequency of 5% to 9.2%. These figures also include surgical revisions and the administration of oral antibiotics [12, 26]. Contrary to current literature, the screw removals in this study were performed without antibiotic prophylaxis [12, 16]. The recorded postoperative wound erythema was 11.4%. However, no antibiotic therapy or surgical revision was required. Nevertheless, these represent complications that could potentially have been avoided with antibiotic prophylaxis.

The infrastructural requirements and associated costs of the method described here are lower, particularly given that ultrasound devices are widely available in medical facilities. Given the absence of a fluoroscopy in many outpatient clinics and hospitals in resource-limited settings, the findings of this study may have significant global applicability. The sonographic method’s portability, lack of radiation exposure, and lower cost may enable safe syndesmotic screw removal in contexts where conventional imaging is unavailable, thereby expanding access to definitive care.

There are several limitations regarding this study. With sonography, only the screw heads can be visualized, while the shaft and threads embedded in the bone cannot be assessed. Consequently, a broken screw cannot be detected. Furthermore, this is a ‘single-center study’ with limited patient diversity. The lack of randomization introduces a selection bias that may impact the results. Another methodological flaw is the absence of inter-rater reliability, which undermines the robustness of the primary outcome. The procedures were performed by residents with varying levels of experience, which influenced both the duration of the operation and the diagnostic accuracy of the ultrasound measurements. The latter is highly examiner-dependent and is also affected by the extent of the soft tissue mantle.

## Conclusions

Ultrasound-guided localization and removal of syndesmotic screws has demonstrated comparable diagnostic accuracy to fluoroscopy-guided removal. The procedure can be performed within a similar operative time. Additional benefits include lower infrastructural requirements and associated costs. Furthermore, this method eliminates radiation exposure, making it a feasible and safe alternative for removing syndesmotic screws.

## Data Availability

The datasets used and/or analysed during the current study are available from the corresponding author on reasonable request.
